# Neoadjuvant Chemotherapy Can Effectively Avoid Unnecessary Extended Resection for Gastric Cancer with Clinical Evidence of Duodenum or Pancreas Head Involvement

**DOI:** 10.7150/jca.105534

**Published:** 2025-01-13

**Authors:** Qianna Jin, Jiaqing Cao, Guobin Wang, Nan He

**Affiliations:** 1 Department of Radiology, Hubei Province Key Laboratory of Molecular Imaging, Union Hospital, Tongji Medical College, Huazhong University of Science and Technology, 1277 JieFang Avenue, Wuhan 430022, China.; 2 Department of Gastrointestinal Surgery, The Second Affiliated Hospital of Nanchang University, 1 MingDe Road, Nanchang 330001, China.; 3 Department of Gastrointestinal Surgery, Union Hospital, Tongji Medical College, Huazhong University of Science and Technology, 1277 JieFang Avenue, Wuhan 430022, China.; 4 Department of Digestive Oncology Surgery, Union Hospital, Tongji Medical College, Huazhong University of Science and Technology, 1277 JieFang Avenue, Wuhan 430022, China.

**Keywords:** gastric cancer, overall survival, prognostic factors, pancreaticoduodenectomy, neoadjuvant chemotherapy

## Abstract

**Purpose:** This study aims to compare the efficacy of two treatment strategies for gastric cancer with clinical evidence of pancreatic head or duodenal involvement: gastrectomy combined with pancreaticoduodenectomy (GPD) and neoadjuvant chemotherapy followed by surgery (NCS).

**Methods:** A retrospective analysis of patient data from January 2012 to January 2022 was conducted to evaluate the outcomes of these two treatment strategies.

**Results:** The study included 284 patients, comprising 78 in the GPD group and 206 in the NCS group. In the NCS group, 119 patients required extended pancreaticoduodenectomy, a significantly smaller proportion compared to the GPD group (p < 0.001). The NCS group successfully avoided unnecessary extended pancreaticoduodenectomy. In contrast, 15 patients in the GPD group underwent surgery despite postoperative pathological confirmation of no pancreatic head or duodenal involvement (p < 0.001). The incidence of Clavien-Dindo grade ≥ IIIb complications was significantly greater in the GPD group than in the NCS group (10.3% vs. 3.3%, p = 0.034). Overall survival was significantly longer in the NCS group, with a median of 25 months compared to 20 months in the GPD group (p = 0.0005). Multivariate Cox regression analysis revealed that tumor diameter ≥7 cm and N3 stage were independent adverse prognostic factors.

**Conclusion:** Neoadjuvant chemotherapy is recommended for patients with gastric cancer presenting clinical evidence of pancreatic head or duodenal involvement. This approach reduces unnecessary extended surgeries, lowers complication rates, and improves overall survival.

## Introduction

Gastric cancer (GC) is a prevalent malignancy in China, characterized by a high prevalence of advanced-stage diagnoses and a 5-year overall survival rate of less than 20% 1-3. The mainstay treatment for GC is surgical excision, aimed at achieving complete tumor removal and thorough lymph node dissection 4.

However, the optimal treatment approach for patients with gastric cancer presenting clinical evidence of pancreatic head or duodenal involvement remains controversial due to the challenges associated with radical resection in these cases 5. While gastrectomy combined with pancreaticoduodenectomy can potentially improve survival by achieving R0 resection, its application is constrained by the high risk of postoperative complications and mortality rates 6-11. Conversely, neoadjuvant chemotherapy has been proposed as a strategy to downstage tumors, increase R0 resection rates, and improve overall survival for advanced GC. However, its effectiveness in managing GC with pancreatic head or duodenal involvement remains inconclusive 12-15.

This study aims to address this gap by comparing the short-term and long-term outcomes of neoadjuvant chemotherapy followed by surgery (NCS) versus gastrectomy combined with extended pancreaticoduodenectomy (GPD). Using retrospective data from Wuhan Union Hospital and the Second Affiliated Hospital of Nanchang University, this analysis evaluates the efficacy of these two treatment strategies in managing GC with clinical evidence of pancreatic head or duodenal involvement.

## Patients and Methods

This study was approved by the institutional ethics committee in accordance with the Declaration of Helsinki (Memo number: 2022LSZ0788) and registered at the Chinese Clinical Trials Registry (ChiCTR2200063959). Anonymized data were used, and informed consent was waived by the ethics committee due to the study's retrospective nature. Data regarding patients' clinicopathological characteristics, surgical details, postoperative complications, and follow-up outcomes were collected and analyzed following the STROCSS reporting guidelines 16.

### Patients

The study included patients who met the following criteria:

- A confirmed diagnosis of gastric cancer;

- Clinical evidence of gastric cancer involving the pancreatic head or duodenum, as defined by multi-detector computed tomography (MDCT), endoscopic ultrasound (EUS), or laparoscopic/open surgical exploration (details provided below);

- Absence of distant metastasis or synchronous malignancies;

- Availability of comprehensive clinical records with follow-up information;

- No severe comorbidities precluding surgery;

- Receipt of the SOX (oxaliplatin/S-1) neoadjuvant chemotherapy regimen;

-Undergoing extended resection (gastrectomy plus pancreatoduodenectomy) or gastrectomy alone.

**Figure 1:**
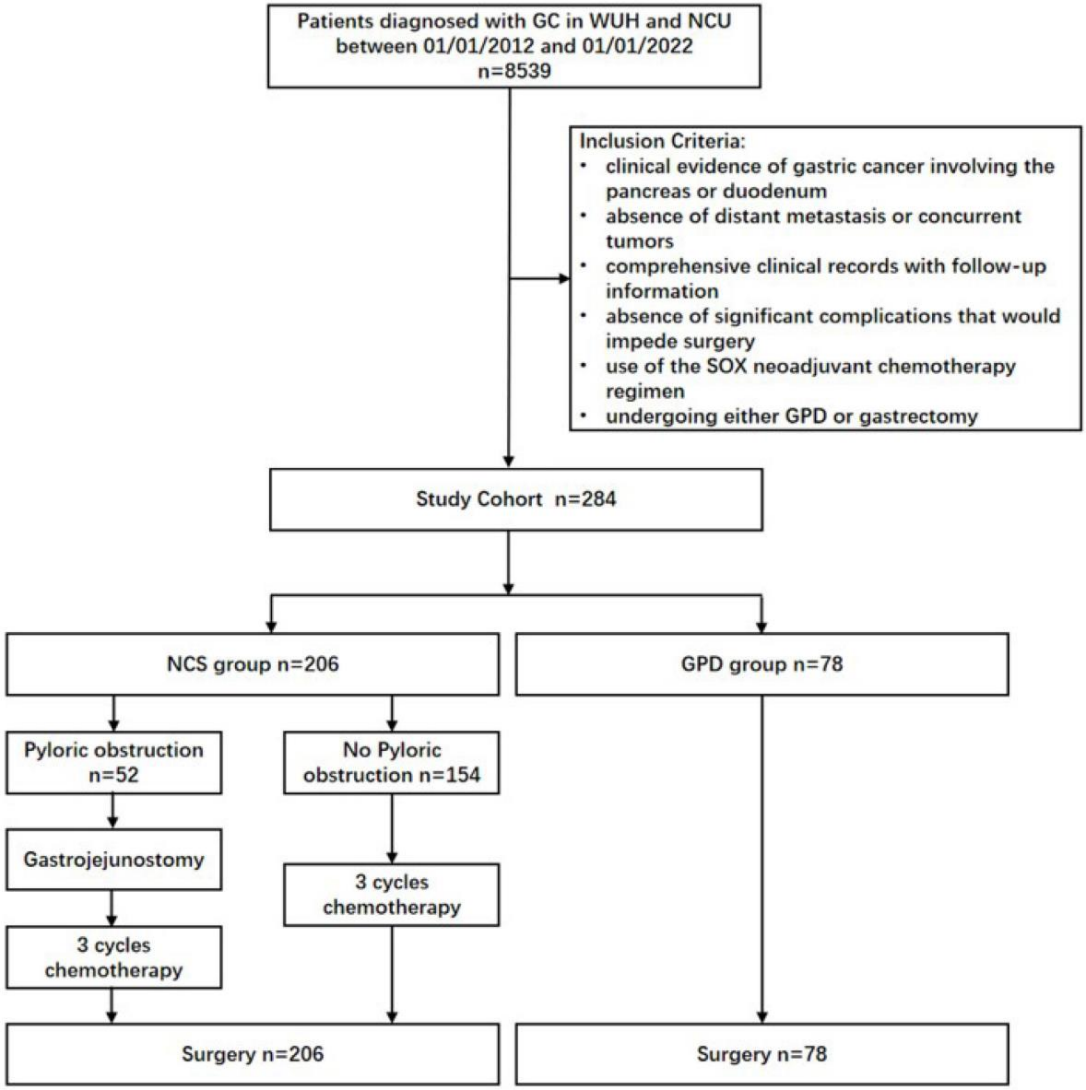
** CONSORT diagram.** NCS, neoadjuvant chemotherapy followed by surgery. GPD, gastrectomy combined with pancreaticoduodenectomy.

Tumor staging was based on the 8th edition of the American Joint Committee on Cancer (AJCC) TNM classification 17. From January 2012 to January 2022, a total of 8,539 patients with gastric cancer were treated at Wuhan Union Hospital and the Second Affiliated Hospital of Nanchang University. Among them, 284 patients met the inclusion criteria. Based on whether they received SOX-based neoadjuvant chemotherapy, 78 patients were assigned to the GPD group, and 206 to the NCS group (Fig. 1).

**Figure 2:**
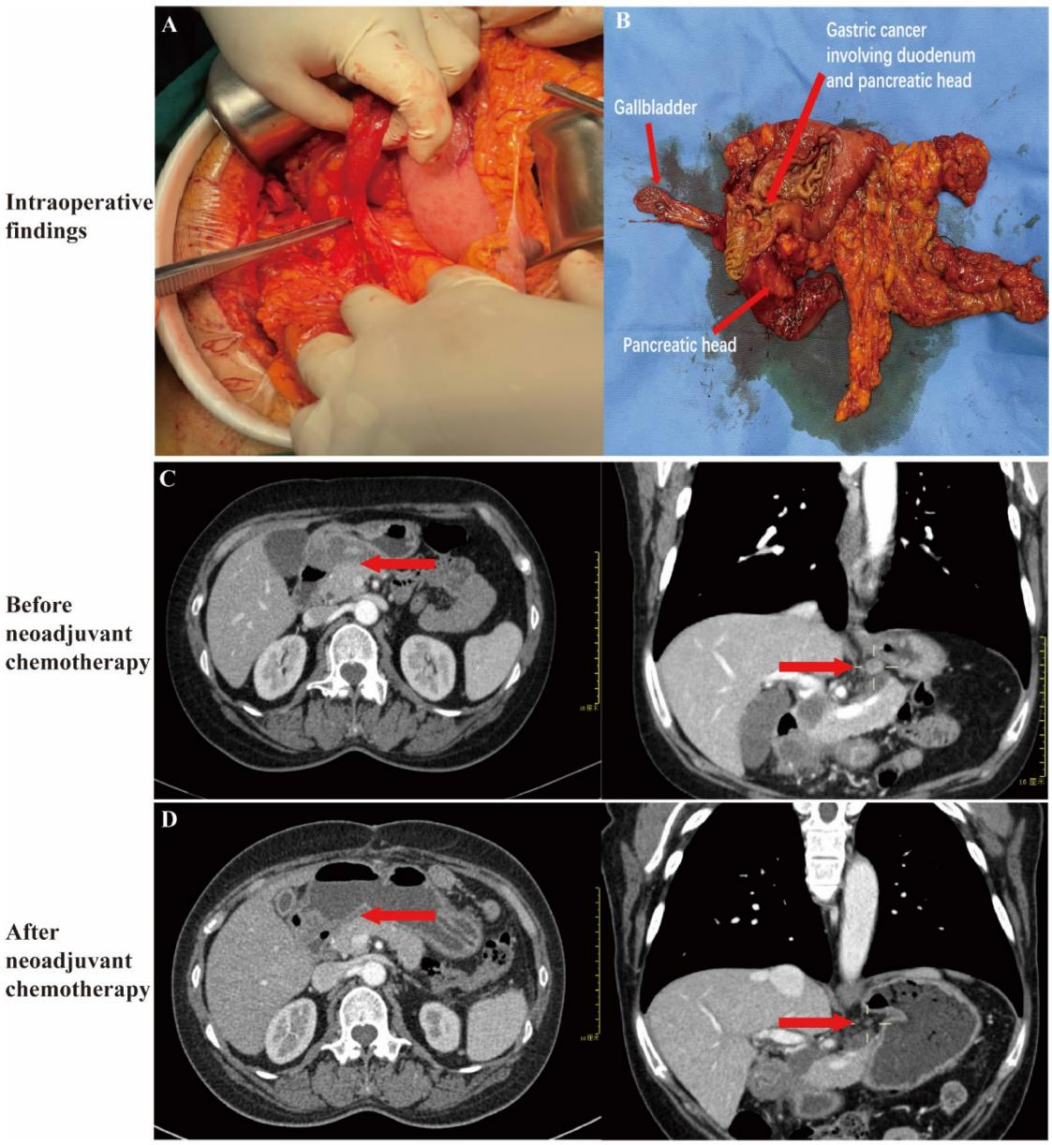
** Intraoperative Findings and Preoperative CT Scan. (A)** Intraoperative findings and **(B)** the resected specimen revealed gastric cancer involving the duodenum and pancreatic head. **(C)** CT scans showed gastric cancer involving the pancreatic head and enlarged lymph nodes prior to neoadjuvant chemotherapy. The red arrows indicate the loss of fat space between the pancreatic head and stomach, as well as the presence of enlarged lymph nodes. **(D)** Post-neoadjuvant chemotherapy CT scans demonstrated significant shrinkage of the gastric cancer lesions and lymph nodes. The red arrows highlight the reappearance of clear fat space between the pancreatic head and stomach and the reduction in lymph node size.

Clinical evidence of locally advanced gastric cancer invading the duodenum or pancreatic head was determined using preoperative multi-detector computed tomography (MDCT) or endoscopic ultrasound (EUS), which showed blurring or disappearance of the fat plane between the gastric cancer lesion and the pancreas head/duodenum 18, 19. Laparoscopic or open surgical exploration revealed adherence of the lesion to the serosal tissue of the duodenum or pancreas, with indistinct borders and restricted mobility (Fig. 2 A and B).

### Perioperative adjuvant therapy

Neoadjuvant chemotherapy was administered using the SOX regimen (oxaliplatin/S-1) following the guidelines of the Chinese Society of Clinical Oncology (CSCO) 1. Tumor response after three cycles of neoadjuvant chemotherapy was assessed using MDCT based on the Response Evaluation Criteria in Solid Tumors (RECIST 1.1) (Fig 2 C and D) 20. In the NCS group, surgery was typically scheduled 4 to 6 weeks after the completion of neoadjuvant chemotherapy, with minor variations based on individual patient recovery and response. Tumor regression grade (TRG) was evaluated postoperatively, and categorized as: Grade 0 (complete response), Grade 1 (viable tumor cells ≤ 1-2%), Grade 2 (viable cells ≤ 50%), or Grade 3 (viable cells > 50%) 21.

Postoperative chemotherapy was given using the same regimen as the preoperative treatment. In cases of recurrence, chemotherapy regimens were adjusted by oncologists as needed. Additionally, six patients from NCS group received postoperative concurrent chemoradiotherapy with consistent dosage levels as per previous studies 22.

### Surgical Procedure

All surgical procedures were performed by surgeons with more than 10 years of experience. Based on intraoperative findings of tumor invasion into the pancreatic head or duodenum, surgeons performed either gastrectomy combined with pancreaticoduodenectomy (GPD) or gastrectomy alone with D2/D2+ lymph node dissection. In the NCS group, 52 patients with pyloric obstruction underwent gastrojejunostomy before initiating neoadjuvant chemotherapy.

### Evaluation and Follow up

The evaluation of clinical and histopathological tumor response followed the criteria established by the Chinese Society of Clinical Oncology (CSCO) and the National Comprehensive Cancer Network (NCCN) 1, 23. Data on clinicopathological factors (e.g., age, gender, BMI, ASA score, preoperative blood albumin, hemoglobin, and CEA levels, tumor diameter, N stage, histological classification, lymphovascular invasion, and neural invasion) and surgical outcomes (e.g., surgical approach, operative duration, blood loss, complications, and mortality) were analyzed. Preoperative data (CEA, blood albumin, and hemoglobin) were obtained during routine examinations, with values for the NCS group collected following neoadjuvant chemotherapy. Postoperative data (tumor diameter, N stage, histological classification, lymphovascular invasion, neural invasion) were derived from pathological findings. Complications were categorized using the modified Clavien-Dindo classification, and postoperative mortality was defined as death within 30 days of surgery. Patients were followed up every three months during the first year, every six months during the second year, and annually thereafter. Follow-up data were collected from medical records, outpatient visits, and telephone interviews until death or December 31, 2023. Overall survival was defined as the duration from surgery to death due to any cause.

### 2.5 Statistical Analysis

Continuous variables were expressed as means or medians, and non-parametric comparisons were performed using the Mann-Whitney U test. Categorical variables were analyzed using Fisher's exact test or Pearson's χ^2^ test. Overall survival was estimated using the Kaplan-Meier method, and multivariate analyses were conducted using Cox's proportional hazard model. A p-value <0.05 was considered statistically significant. All statistical analyses were performed using SPSS 22.0 software (SPSS Inc., Chicago, IL, USA).

## Results

### 3.1 Clinicopathological characteristics

Table 1 summarizes the clinicopathological characteristics of the patients. No significant differences were observed between the NCS and GPD groups in terms of age, gender, ASA score, BMI, or receipt of postoperative adjuvant therapy (p > 0.05). However, preoperative serological analysis revealed a significantly higher CEA level in the GPD group compared to the NCS group (19.42 ± 3.96 vs. 6.36 ± 0.67 μg/L, p < 0.0001). Preoperative hemoglobin and albumin levels were comparable between the groups (p > 0.05).

Postoperative pathological findings analysis showed no significant differences in N stage, histological classification, lymphovascular invasion, or nerve invasion between the groups (p > 0.05). However, tumor size was significantly smaller in the NCS group than in the GPD group (5.97 ± 1.38 cm vs. 7.51 ± 1.48 cm, p < 0.0001). Additionally, 155 patients in the NCS group achieved a tumor regression grade (TRG) of ≤ 2 following neoadjuvant chemotherapy, with 36 achieving a TRG of 0 21.

### 3.2 Short-Term Outcomes of Surgery

Table 2 outlines the short-term surgical outcomes. In the GPD group (n = 78), all patients underwent gastrectomy combined with extended pancreaticoduodenectomy. In the NCS group (n = 206), 119 patients received extended pancreaticoduodenectomy, while the remaining 87 underwent gastrectomy alone. Neoadjuvant chemotherapy significantly reduced the need for extended pancreaticoduodenectomy (57.7% vs. 100%, p < 0.001).

Pathological examination revealed no evidence of duodenal or pancreatic invasion in surgical specimens from 15 patients in the GPD group, whereas all 119 patients in the NCS group who underwent extended pancreaticoduodenectomy exhibited confirmed tumor infiltration (80.8% vs. 100%, p < 0.001). Furthermore, all 87 patients in the NCS group who underwent gastrectomy alone achieved R0 resection, indicating no residual tumors. This underscores the efficacy of neoadjuvant chemotherapy in reducing the need for unnecessary extensive resections.

The NCS group demonstrated significantly shorter operation times (230.2 ± 46.86 minutes vs. 270.4 ± 57.23 minutes, p < 0.001) and reduced intraoperative blood loss (253.4 ± 142.2 mL vs. 394.3 ± 94.02 mL, p < 0.001) compared to the GPD group. Postoperative complications, such as intra-abdominal hemorrhage, pancreatic fistula, and biliary fistula, were more frequent in the GPD group (p < 0.05). Severe complications (Clavien-Dindo grade ≥ IIIb) occurred in 15 cases: 7 in the NCS group and 8 in the GPD group (3.3% vs. 10.3%, p = 0.034). These included 6 cases requiring intervention for bleeding, 3 cases of refractory anastomotic leakage necessitating surgery, and 6 cases of treatment-related mortality. Among treatment-related deaths, 2 cases were caused by multiple-organ failure due to anastomotic leakage, 1 case by pulmonary embolism, and 3 cases by fatal bleeding resulting from pancreatic fistula.

### Overall Survival and Prognostic Factors

**Figure 3:**
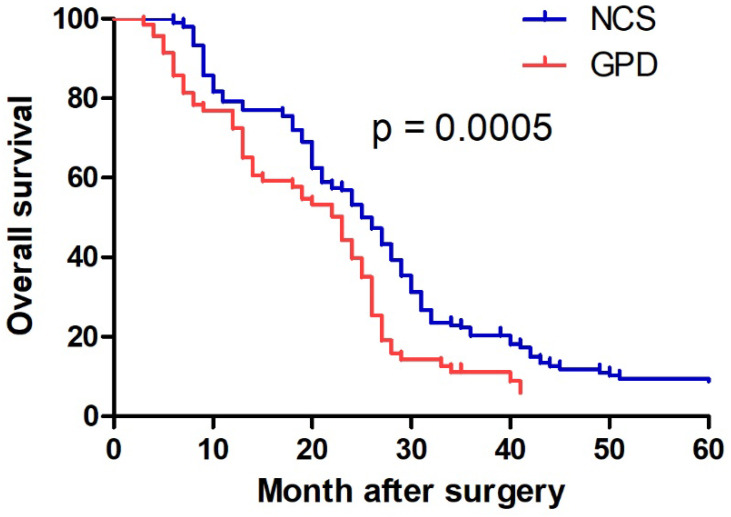
** Kaplan-Meier estimated overall survival.** The red and blue lines indicated the GPD and NCS group, respectively. NCS, neoadjuvant chemotherapy followed by surgery. GPD, gastrectomy combined with pancreaticoduodenectomy.

Patients in the NCS group exhibited significantly longer overall survival than those in the GPD group (median: 25 months vs. 20 months, p = 0.0005) (Fig. 3). Univariate analysis identified tumor diameter, N stage, and treatment strategy as significant factors influencing survival (Table 3). Multivariate Cox regression analysis determined tumor diameter ≥7 cm (p = 0.015) and N3 stage (p = 0.001) as independent poor prognostic factors (Table 3). However, treatment strategy (NCS or GPD) was not independently associated with prognosis (p = 0.08).

## Discussion

Preoperative diagnosis of gastric cancer infiltration into the duodenum or pancreatic head remains highly challenging. Studies have reported that the accuracy of computed tomography (CT) and endoscopic ultrasonography (EUS) in detecting tumor extension to adjacent organs is less than 50% 12, 24. Even surgical exploration often fails to definitively determine whether the tumor has invaded the duodenum or pancreas. Postoperative pathological findings analyses indicate that over one-third of patients who undergo extended organ resection for locally advanced gastric cancer lack confirmed tumor invasion 25-27. In this study, pathological analysis of specimens from 15 patients in the GPD group revealed no evidence of duodenal or pancreatic head invasion.

Intraoperative features such as indistinct boundaries between the tumor or lymph nodes and the pancreas, adhesions to adjacent organs, or thickened duodenal walls may suggest tumor infiltration. However, these characteristics can also result from localized inflammation, intestinal wall thickening due to edema, or fibrous tissue proliferation, complicating the differentiation between malignant infiltration and benign processes. Consequently, some patients may undergo unnecessary extended pancreaticoduodenectomy.

Neoadjuvant chemotherapy appeared to address this issue by significantly reducing tumor size in the NCS group compared to the GPD group. This reduction likely attenuated the local inflammatory response, enabling more precise intraoperative assessment of tumor infiltration. In the NCS group, 119 patients required extended pancreaticoduodenectomy, all of whom exhibited confirmed tumor infiltration upon pathological examination. Meanwhile, the remaining 87 patients were able to avoid unnecessary extended surgery, achieving R0 resection without residual tumors. These findings supported the recommendation of neoadjuvant chemotherapy for gastric cancer patients with clinical evidence of pancreatic head or duodenal involvement, consistent with previous studies 6, 9, 28.

The GPD group experienced significantly greater intraoperative blood loss and longer surgical durations compared to the NCS group. Previous studies have consistently shown higher rates of complications, including pancreatic, biliary, and anastomotic leaks, following extended pancreaticoduodenectomy for locally advanced gastric cancer 29-34. This increased complication rate is largely attributed to the broader scope and technical complexity of the procedure compared to gastrectomy. Consequently, postoperative complications, particularly those classified as Clavien-Dindo grade ≥ IIIb, are more frequent after extended pancreaticoduodenectomy.

The primary efficacy endpoint of this study was overall survival. Patients in the NCS group demonstrated significantly longer overall survival compared to those in the GPD group. Multivariate Cox regression analysis identified tumor diameter ≥7 cm and N3 stage as independent poor prognostic factors. Previous research has shown that neoadjuvant chemotherapy effectively reduces tumor size, down-stages the tumor, and increases the R0 resection rate in patients with locally advanced gastric cancer 28. Additionally, N stage is a critical prognostic indicator, with an N3 stage associated with a 5-year survival rate of only 21.3%, even after achieving R0 resection 35. Studies have also indicated that patients undergoing extended pancreatoduodenectomy achieved an R0 resection, resulting in a 3-year survival rate of 34-47.6%, significantly higher than the 20.4% observed in patients undergoing gastric tumor resection alone 8, 9, 36. Neoadjuvant chemotherapy can increase R0 resection rate to 79-87% and improve 5-year survival rate to 36-38% without increasing postoperative complications or mortality 14, 15, 37. Therefore, neoadjuvant chemotherapy is strongly recommended for gastric cancer with clinical evidence on pancreatic or duodenal involvement to reduce complication rates and improve overall survival.

This study has several limitations. First, its retrospective design relies on the accuracy and completeness of the collected data, which may introduce inherent biases. Second, the inclusion of patients from only two institutions limits the generalizability of the findings due to potential selection bias. Third, the absence of a universally reliable preoperative staging method for locally advanced gastric cancer involving the pancreatic head or duodenum remains a significant challenge. Commonly recommended staging techniques, including MDCT, EUS, and surgical exploration, are associated with risks of misdiagnosis or missed diagnoses, potentially biasing the results. To address these limitations, future research should prioritize prospective, multi-center studies with larger sample sizes to generate more robust and generalizable evidence.

In conclusion, for gastric cancer with clinical evidence of the duodenum or pancreas head involvement, our findings demonstrate that neoadjuvant chemotherapy significantly reduces the need for unnecessary extended resections, lowers the incidence of postoperative complications, and improves overall survival rates. Therefore, neoadjuvant chemotherapy should be recommended for these patients.

## Figures and Tables

**Table 1:** Patients' clinicopathological characteristics

	NCS (n=206)	GPD (n = 78)	P
**Mean age (SD)**	59.51±9.19	57.58±13.43	0.17
**Gender**			0.16
Male	162	55	
Female	44	23	
**ASA score (range)**			0.59
1-2	129	46	
3	77	32	
**Preoperative BMI (kg/m2)**	25.54±4.18	26.36±6.03	0.2
**Preoperative serum**			
Albumin (g/dl), mean ± SD	37.91±4.39	37.46±4.02	0.44
Hemoglobin (g/dl), mean ± SD	110.1±14.63	110.4±17.64	0.89
CEA(μg/L), mean ± SD	6.36±0.67	19.42±3.96	< 0.0001
**Tumor diameter (cm)**	5.97±1.38	7.51±1.48	< 0.0001
**Histological classification**			0.079
Poorly differentiated	152	49	
Well-moderately differentiated	54	29	
**Neural invasion**			0.54
NO	152	61	
YES	54	17	
**Lymphovascular invasion**			0.13
NO	156	52	
YES	50	26	
**Pathological N stage**			0.36
N0~2	159	56	
N3	47	22	
**Postoperative adjuvant therapy**			0.19
Chemotherapy	200	78	
Chemoradiotherapy	6	0	

SD: standard deviation; ASA: American Society of Anesthesiologists; NCS: neoadjuvant chemotherapy plus surgery; GPD: gastrectomy plus pancreaticoduodenectomy.

**Table 2:** Short-term outcomes of surgery

	NCS (n=206)	GPD (n = 78)	P
**Surgical treatment**			< 0.001
gastrectomy with pancreaticoduodenectomy	119	78	
gastrectomy alone	87	0	
**Unnecessary extended resection**			< 0.001
Yes	0	15	
No	119	63	
**Blood loss (ml)**	253.4±142.2	394.3±94.02	< 0.001
**Duration of surgery (min)**	230.2±46.86	270.4±57.23	< 0.001
**Complications**			
Gastroparesis	10	2	0.52
Anastomotic fistula	15	3	0.42
Abdominal hemorrhage	5	7	0.021
Gastrointestinal hemorrhage	4	4	0.22
Disruption of wound	9	8	0.089
Biliary fistula	2	6	0.0063
Pancreatic fistula	5	10	0.0013
Pulmonary infection	5	4	0.26
**Clavien-Dindo score ≥ IIIb**	7	8	0.034
**Treatment-related mortality**	3	3	0.35

NCS: neoadjuvant chemotherapy plus surgery; GPD: gastrectomy plus pancreaticoduodenectomy.

**Table 3:** Cox proportional hazard regression for overall survival in gastric cancer with duodenum or pancreatic head invasion patients

Variable	Univariate analysis			Multivariable analysis			
	OR	95% CI	P	OR		95% CI	P
**Ages**							
> 65 vs ≤ 65	1.64	0.9173-2.945	0.10				
**ASA**							
≥ 3 vs < 2	0.01	0.27- 1.73	0.90				
**Hemoglobin**							
≤90g/l vs >90g/l	1.19	0.82- 1.73	0.37				
**CEA**							
≥5u/l vs <5u/l	0.84	0.63-1.12	0.24				
**Albumin**							
<35g/l vs ≥35g/l	0.89	0.66-1.20	0.46				
**Tumor diameter**							
≥7cm vs <7cm	0.52	0.37- 0.73	< 0.0001		1.52	1.08-2.13	0.015
**Histological classification**							
Poorly vs Well-moderately differentiated	0.78	0.58-1.04	0.10		1.24	0.94-1.63	0.12
**Lymphovascular invasion**							
with vs without	1.15	0.84-1.59	0.38		1.02	0.76-1.37	0.88
**Neural invasion**							
with vs without	0.90	0.65-1.23	0.49		1.16	0.87-1.55	0.3
**Surgical margin**							
R1 vs R0	0.86	0.56-1.34	0.51		1.27	0.84-1.93	0.26
**Pathological N stage**							
N3 vs <N0~2	8.78	0.39-0.83	0.0031		1.69	1.23-2.31	0.001
**Treatment strategy**							
NCS vs GPD	0.57	0.40-0.80	0.0014		0.73	0.53-1.03	0.08
							

OR: odds ratio; CI: confidence interval; NCS: neoadjuvant chemotherapy plus surgery; GPD: gastrectomy plus pancreaticoduodenectomy.
